# Postmortem Gene Expression and the Thanatotranscriptome: Postmortem Interval Estimation, Forensic Applications, and Future Perspectives

**DOI:** 10.3390/ijms27209036

**Published:** 2026-10-12

**Authors:** Özlem Akpınar, Ziyaettin Erdem, Fettah Zeki Keskiner, Kenan Kaya

**Affiliations:** 1 Eskişehir Branch Directorate, Council of Forensic Medicine, Eskişehir 26030, Türkiye; zyferdem@gmail.com; 2 Adana Group Directorate, Council of Forensic Medicine, Adana 01060, Türkiye; fz_keskiner80@hotmail.com; 3 Department of Forensic Medicine, Çukurova University, Adana 01330, Türkiye; k_kaya_71@hotmail.com

**Keywords:** thanatotranscriptome, postmortem interval, postmortem gene expression, mRNA, miRNA, RNA degradation, transcriptomics, forensic medicine, forensic pathology

## Abstract

Postmortem molecular changes are more complex than a passive, uniform cessation of all cellular activity. Human and animal studies have demonstrated that certain RNA transcripts remain detectable for defined periods after death and that transcript levels exhibit tissue- and time-dependent changes. These postmortem RNA dynamics underpin the concept of the thanatotranscriptome and may provide molecular information relevant to postmortem interval (PMI) estimation. Postmortem changes have been reported in the abundance of transcripts associated with apoptosis, cellular stress, immune responses, and DNA damage response and repair pathways. However, observed transcript levels may reflect the combined effects of antemortem RNA stability, selective RNA degradation, and potentially residual transcriptional activity. A longitudinal study of human blood reported a root mean square error (RMSE) of 4.75 h for a PMI estimation model based on multiple transcripts, illustrating the quantitative potential of this approach. Nevertheless, RNA profiles may be influenced by tissue type, temperature, cause of death and agonal processes, interindividual biological variability, and sampling and storage conditions. Methodological heterogeneity and the lack of independent external validation further limit the translation of these approaches into routine forensic practice. This review critically evaluates the molecular basis of postmortem gene expression and RNA dynamics, messenger RNA (mRNA) and microRNA (miRNA) biomarkers, transcriptome-based models for PMI estimation, and their potential forensic applications, with particular emphasis on the need for standardization, independent validation, and multi-biomarker approaches.

## 1. Introduction

Accurate estimation of the time elapsed since death remains one of the fundamental and persistent challenges in forensic medicine and forensic pathology. The interval between death and the discovery of the body or subsequent forensic examination is commonly defined as the postmortem interval (PMI). Estimation of the PMI is particularly important for the chronological reconstruction of events in suspicious and medicolegal deaths. Reliable PMI estimation may contribute to establishing the timeline of death-related events, evaluating statements and other evidence obtained during an investigation, and, in certain cases, determining the temporal relationship between suspects and the event. However, postmortem changes are influenced by environmental conditions, individual characteristics, and death-related factors, making highly accurate PMI estimation challenging [1,2].

Conventional approaches to PMI estimation include the assessment of early postmortem changes, such as body cooling, rigor mortis, and postmortem lividity, as well as various physical, biochemical, and morphological methods. Forensic entomological approaches may also be applied at more advanced postmortem stages. However, the performance of these methods is affected by environmental and individual factors, and no single method provides consistently high accuracy across the entire postmortem period [1,2]. Therefore, PMI assessment requires a multiparametric approach in which the available findings are interpreted collectively.

The limitations of conventional methods have prompted increasing interest in identifying more objective and quantifiable molecular biomarkers associated with PMI. In this context, RNA stability, RNA degradation, and time-dependent changes in postmortem gene expression have been increasingly investigated as potential molecular indicators of PMI [3,4,5]. RNA-based markers and postmortem gene expression have attracted particular attention because of their potential to reflect time- and tissue-dependent molecular changes after death [3].

RNA is a more labile molecule than DNA and is susceptible to degradation during the postmortem period. Nevertheless, human and animal studies have demonstrated that certain RNA transcripts remain detectable for defined periods after death and that RNA levels may exhibit time-, tissue-, and transcript-dependent changes [3,6,7,8]. Large-scale analyses of human tissues have further demonstrated that postmortem transcriptomic changes are markedly tissue-specific and cannot be explained solely by a uniform process of RNA degradation [7,9]. These findings have provided a basis for investigating RNA stability and time-dependent changes in transcript abundance as potential molecular indicators for PMI estimation.

These advances have promoted the systematic investigation of postmortem RNA dynamics and contributed to the development of the concept of the thanatotranscriptome. The thanatotranscriptome broadly refers to the postmortem transcriptomic profile comprising RNA transcripts detectable in cells and tissues after death [3,6]. This concept encompasses not only postmortem RNA stability and degradation but also increases and decreases in the abundance of specific transcripts after death and the biological processes associated with these changes [3,6,7,8].

Recent studies indicate that thanatotranscriptomic research is expanding beyond targeted mRNA analyses toward broader transcriptome-wide approaches. In a recent study of human prostate tissue, total RNA sequencing was used to investigate PMI-associated changes in messenger RNA (mRNA) and long non-coding RNA (lncRNA) expression, as well as alternative splicing patterns [10]. This approach demonstrates that postmortem RNA research is not limited to the quantification of individual transcripts but may also include the investigation of transcriptome-wide expression and RNA-processing patterns relevant to forensic applications.

Current evidence suggests that specific RNA transcripts and multi-transcript profiles may be associated with PMI and that quantitative models for PMI estimation can be developed from transcriptomic data [7,9,11,12]. However, postmortem RNA profiles may be influenced by tissue type, environmental conditions, cause of death and agonal processes, interindividual biological variability, sampling and storage conditions, and the analytical and computational methods employed [4,5,9]. Heterogeneity in tissue and sample types, experimental conditions, analytical platforms, normalization strategies, and statistical approaches across postmortem RNA and transcriptomic studies limits both the comparability of findings and the generalizability of resulting PMI models [4,5]. Therefore, the forensic potential of thanatotranscriptomics depends not only on identifying PMI-associated transcripts but also on systematically accounting for these sources of variability and validating predictive models in independent sample sets.

The aim of this review is to provide a comprehensive and critical evaluation of the current evidence on postmortem gene expression and RNA dynamics within the conceptual framework of the thanatotranscriptome. Specifically, the review addresses postmortem RNA stability and degradation, transcriptomic changes observed across different tissues and biological samples, the potential biological mechanisms underlying these changes, mRNA- and miRNA-based biomarkers, and transcriptome-based approaches to PMI estimation. In addition, it examines the potential forensic applications of thanatotranscriptomics beyond PMI estimation, the methodological limitations of current studies, the need for standardization and independent validation, and the requirements for translating these approaches from the research setting into routine forensic practice.

## 2. The Concept of the Thanatotranscriptome

The transcriptome refers to the complete set of RNA transcripts present in a cell, tissue, or organism at a given time. Gene expression profiles may vary according to the physiological state of the cell and in response to intrinsic and extrinsic stimuli. The transcriptome can therefore be regarded as a dynamic molecular profile reflecting the state of cells and tissues at a particular point in time [3].

Following organismal death, postmortem molecular changes do not occur simultaneously or uniformly across all cells and tissues. Furthermore, the postmortem stability and degradation rates of RNA molecules synthesized before death may vary according to RNA type, transcript-specific and tissue-specific characteristics, and postmortem conditions [4,5,7]. Consequently, the postmortem RNA profile represents a dynamic molecular landscape characterized by temporal and tissue-specific variability.

Within this framework, the thanatotranscriptome broadly refers to the postmortem transcriptomic profile comprising RNA transcripts detectable in cells and tissues after the death of an organism [3,6]. Interpretation of this profile requires a clear distinction among four related but conceptually different phenomena: RNA persistence and stability, RNA degradation, differential transcript abundance, and potential postmortem transcriptional activity.

**Postmortem RNA persistence and stability:** Transcripts synthesized before death may remain detectable for variable periods after death. Their persistence depends on transcript-specific and tissue-specific stability and does not require continued RNA synthesis.

**Postmortem RNA degradation:** RNA generally undergoes progressive degradation after death, but degradation rates vary among RNA species, individual transcripts, and tissues. Consequently, postmortem RNA loss is neither uniform nor synchronous.

**Differential transcript abundance:** Increases or decreases in the measured abundance of specific transcripts during the postmortem period represent an observed expression pattern rather than a mechanism in themselves. Such changes may arise from differences in transcript stability and degradation kinetics and therefore cannot, on their own, establish the underlying biological process.

**Potential postmortem transcriptional activity:** Residual or newly initiated transcription after death represents a mechanistically distinct possibility. An increase in transcript abundance alone is insufficient to establish de novo RNA synthesis, because differential stability and degradation may produce similar abundance patterns. Demonstrating ongoing postmortem transcription therefore requires independent experimental evidence capable of distinguishing newly synthesized RNA from pre-existing transcripts. Experimental approaches that directly assess nascent RNA, such as metabolic labeling of newly synthesized transcripts or nascent RNA sequencing, may help distinguish ongoing RNA synthesis from changes attributable to differential RNA stability or degradation; however, their applicability to human postmortem tissues requires specific validation [13,14]. Accordingly, the measured thanatotranscriptome should be understood as the composite postmortem RNA profile resulting from these potentially overlapping processes, rather than as evidence of any single underlying mechanism. This distinction is important for interpreting postmortem transcriptomic changes and for evaluating their potential use as PMI-related biomarkers [4,5,7,9].

## 3. Biological Basis of Changes in Postmortem Gene Expression

Following death, the cessation of circulation and oxygenation leads to tissue hypoxia and ischemia, the disruption of energy metabolism, and the progressive loss of cellular homeostasis. Because tissues differ in their metabolic characteristics and tolerance to ischemia, the molecular consequences of these changes may also vary according to tissue type and postmortem interval [7,9]. Hypoxia, energy depletion, and cellular stress arising during the early postmortem period may influence molecular pathways involved in cell survival and death. Human and animal studies have reported that postmortem transcriptomic changes may involve genes associated with stress responses, immune and inflammatory processes, apoptosis, and DNA damage response and repair pathways [6,7,8,11]. However, changes in the abundance of transcripts associated with these pathways do not, by themselves, demonstrate that the corresponding biological processes remain fully functional after death.

Key experimental evidence regarding postmortem transcriptomic changes comes from the study by Pozhitkov et al., who used zebrafish (*Danio rerio*) and mouse (*Mus musculus*) models [8]. In this study, postmortem transcriptomic changes were monitored for up to 96 h in zebrafish and 48 h in mice, and transcript profiles corresponding to a total of 1063 genes across the two model organisms were reported to show significantly higher postmortem abundance relative to antemortem levels. The temporal distribution of these changes was not uniform: increases in a substantial proportion of transcripts emerged during the early postmortem period, whereas others became more pronounced at later time points [8].

Functional analyses indicated that transcripts showing increased abundance were associated with a range of biological functions, including stress responses, immune and inflammatory processes, apoptosis, cellular transport, developmental processes, and epigenetic regulation [8]. These temporally structured and functionally clustered changes suggest that the postmortem RNA profile may not be attributable solely to uniform and stochastic RNA degradation. Moreover, because these findings were derived from experimental animal models, their generalizability to human postmortem biology and real-world forensic cases remains limited.

Evidence from human studies also indicates that the postmortem transcriptome exhibits tissue- and time-dependent changes. In a large-scale analysis based on data from the Genotype-Tissue Expression (GTEx) project, Ferreira et al. reported that the relationship between postmortem interval and RNA stability was markedly tissue-specific and that postmortem changes in gene expression could not be explained solely by stochastic RNA degradation [7]. In particular, comparison of antemortem and postmortem blood samples demonstrated that certain transcripts exhibited time-dependent patterns of increased or decreased abundance after death [7].

In a longitudinal study of postmortem blood samples obtained from individuals with known times of death, Antiga et al. identified groups of transcripts with distinct temporal patterns, including both increases and decreases in transcript abundance [11]. Pathway analyses indicated that early postmortem changes were associated with cell survival and DNA damage response and repair processes, and the authors interpreted these findings as consistent with regulated postmortem processes that could not be explained solely by passive RNA degradation [11].

Distinguishing transcript stability from degradation dynamics represents a fundamental methodological challenge in the biological interpretation of the postmortem transcriptome. Studies of human tissues have demonstrated that postmortem RNA changes are both tissue- and gene-specific and that certain transcripts may persist longer than others [7,9]. Therefore, the relatively high abundance of a particular transcript during the postmortem period may reflect greater transcript stability or slower degradation relative to other transcripts, in addition to potential ongoing molecular activity.

Taken together, these findings indicate that postmortem transcriptomic changes are biologically heterogeneous and tissue-specific. Time-dependent changes in transcript abundance observed in both experimental animal models and human tissues support the view that the postmortem RNA profile may not be explained solely by passive and uniform degradation [7,8,11]. Nevertheless, elucidating the mechanisms underlying these changes and demonstrating their reproducibility in real-world forensic cases are essential before thanatotranscriptomic findings can be translated into reliable biomarkers for PMI estimation.

## 4. RNA Stability and Postmortem RNA Degradation

RNA stability and degradation kinetics are fundamental components of RNA-based postmortem interval (PMI) research. Owing to its chemical structure, RNA is more susceptible to hydrolytic cleavage than DNA. The 2′-hydroxyl (2′-OH) group of the ribose moiety can, under appropriate conditions, facilitate intramolecular nucleophilic attack on the adjacent phosphodiester bond, thereby contributing to hydrolytic cleavage of the RNA backbone [15]. RNA is also susceptible to enzymatic degradation by ribonucleases. In addition to these intrinsic chemical properties, RNA stability may be influenced by temperature, pH, ionic conditions, RNA secondary structure, and RNA–protein interactions [15].

Although RNA integrity generally decreases over time after death, this process is neither linear nor uniform. Studies of postmortem human tissues have demonstrated that RNA integrity may be influenced by tissue type as well as antemortem and postmortem variables, and that RNA degradation can affect quantitative measurements of gene expression [16]. In a study of postmortem human brain, cardiac muscle, and skeletal muscle samples, Koppelkamm et al. demonstrated that RNA integrity differed among tissues and that reduced RNA integrity could affect the reliability of gene expression measurements obtained by quantitative reverse transcription polymerase chain reaction (RT-qPCR) [16]. Therefore, in postmortem transcriptomic studies, RNA degradation should be considered not only a biological process but also an important preanalytical and analytical variable that may influence measurement outcomes.

Different RNA species, as well as individual transcripts within the same RNA class, may exhibit distinct stability profiles during the postmortem period. Similarly, the degradation kinetics of a given transcript may vary across tissues [4,5,16]. Therefore, a decline in overall RNA integrity and a change in the abundance of a specific mRNA transcript do not represent the same biological phenomenon. In postmortem RNA analyses, overall RNA integrity, transcript-specific abundance, and the normalization strategy employed should therefore be evaluated as distinct, although interrelated, parameters [4,5,16].

Using 2016 high-quality postmortem samples from 316 donors of European ancestry obtained from the GTEx dataset, Zhu et al. systematically investigated PMI-associated gene expression changes across 15 human tissue types and subtypes over a PMI range of 1–27 h [9]. The study demonstrated that PMI-associated expression changes were markedly tissue- and gene-specific and further provided evidence that genotype-by-PMI interactions may influence postmortem expression profiles for certain genes [9]. mRNA profiles in central nervous system tissues were reported to be relatively more stable, whereas more pronounced postmortem changes were observed in some gastrointestinal tissues [9]. These findings indicate that postmortem RNA dynamics are determined not only by elapsed time since death but also by tissue- and transcript-specific characteristics.

This heterogeneity in RNA stability and degradation is critically important for the interpretation of RNA-based PMI estimation in forensic practice. Current evidence does not support the assumption of a universal “RNA degradation clock” applicable across all tissues and transcripts [4,5,9]. Instead, characterizing tissue- and transcript-specific postmortem kinetics and accounting for tissue-specific biological variability in the development of PMI estimation models appear to be a more realistic approach.

Human dental pulp has been investigated as a potential tissue for studying RNA degradation and, in particular, for estimating longer PMIs because of its anatomically protected location. In a pilot study by Poór et al., RNA degradation in human dental pulp samples was assessed using the RNA integrity number (RIN) and PCR products of different lengths following reverse transcription [17]. Time-dependent changes in RNA integrity were reported to provide information relevant to elapsed time during the first 21 days, whereas the PCR-based approach showed potential for assessing broader time intervals over longer incubation periods [17]. However, because the study was conducted under controlled experimental conditions, the generalizability of these findings to real-world forensic cases remains limited.

In a more recent study, Yin et al. investigated the potential of mRNA degradation in human dental pulp for estimating longer PMIs using high-throughput sequencing and RT-qPCR [18]. Five candidate mRNAs—*SRSF5*, *FGFR1*, *ACADVL*, *FOS,* and *LRP1*—were reported to exhibit decreasing abundance over an experimental period of 0–28 days, and mathematical models based on these transcripts were developed [18]. Multi-marker models demonstrated better fit and predictive performance than single-transcript models. However, this study employed an ex vivo model in which teeth extracted from living individuals were maintained under controlled conditions; consequently, time zero represented the time of tooth extraction rather than the biological death of the individual [18]. Thus, although the findings provide important experimental evidence for time-dependent mRNA degradation in human dental pulp, the applicability of the resulting models to real-world postmortem forensic cases requires validation in independent studies.

Recent studies investigating the potential of RNA degradation for PMI estimation have also begun to evaluate multiple RNA species in combination. Gupta et al. examined degradation patterns of mRNA, ribosomal RNA (rRNA), and miRNA in liver, lung, and heart tissues using RT-qPCR and developed tissue-specific multivariable models for time estimation [19]. In addition to mRNA transcripts such as *GAPDH*, *ACTB*, *RPS10*, and *RPS29*, the study evaluated 5S, 18S, and 28S rRNA as well as tissue-associated miRNAs. The explanatory performance of the multiple regression models varied across tissues, with the highest coefficient of determination (R^2^) reported for the heart (0.75), followed by the liver (0.739), and a lower value for the lung (0.57) [19]. These findings support the potential of RNA degradation to provide quantitative information relevant to PMI while also demonstrating that model performance depends on tissue type and the RNA markers included.

The systematic review by Thakral et al., which evaluated the role of RNA stability and degradation across different tissues in PMI estimation, demonstrated that findings regarding postmortem RNA behavior are not entirely consistent across studies [5]. Major limitations identified included the substantial proportion of available evidence derived from non-human models, the relatively limited number of human studies, and the potential effects of temperature and agonal factors on RNA degradation. The review also emphasized the importance of tissue selection in RNA-based PMI assessment and highlighted the need to investigate approaches integrating multiple molecular parameters [5].

In a 2024 systematic review, Cianci et al. evaluated the available evidence on mRNA-based approaches to PMI estimation and included 21 studies [4]. Although frequently investigated transcripts such as *ACTB* and *GAPDH* were reported to show potential, particularly during relatively short postmortem intervals, the review emphasized that the postmortem stability of transcripts used as reference genes cannot be assumed a priori [4]. The predominant focus on relatively short postmortem intervals, methodological heterogeneity, lack of standardization, limitations of existing mathematical models, and inconsistent reporting of prediction errors were identified as major limitations of the current literature [4].

Among the variables affecting postmortem RNA degradation, temperature is particularly important. In addition, tissue type, cause of death and agonal processes, the timing and method of sampling, storage conditions, RNA isolation and quantification methods, and normalization strategies may influence the measured postmortem RNA profile [4,5,9,16]. These sources of variability may contribute to differences in the degradation kinetics of the same RNA marker across studies and may reduce the performance of PMI models when applied to independent sample sets.

Accordingly, changes in RNA abundance or integrity measured in a postmortem sample should not be interpreted solely as direct indicators of the time elapsed since death. Current evidence suggests that approaches characterizing tissue- and transcript-specific kinetics and integrating multiple RNA markers are more realistic than assuming a universal model of RNA degradation [5,9,17,18,19]. For RNA-based PMI models to be translated into forensic practice, temperature and sampling conditions should be standardized, appropriate normalization strategies should be applied, prediction error and uncertainty should be explicitly reported, and models should be validated in independent cohorts comprising real-world forensic cases [4,5,16,17,18,19].

## 5. The Thanatotranscriptome Across Different Tissues

### 5.1. Blood

Blood is an important biological material for thanatotranscriptomic research because it is frequently available in forensic investigations and enables the assessment of postmortem molecular changes. Early studies suggested that RNA degradation and transcript-specific expression changes in postmortem blood may be associated with PMI [20,21]. Bauer et al. reported an association between mRNA degradation and PMI [20], whereas González-Herrera et al. found no significant correlation between the RNA integrity number (RIN) and PMI but identified differences in the expression of specific transcripts across PMI groups [21]. These findings highlight the importance of distinguishing overall RNA integrity from transcript-specific postmortem changes.

Larger-scale transcriptomic analyses have demonstrated that molecular changes in postmortem blood are more complex than a simple and uniform process of RNA degradation. Ferreira et al. compared RNA-sequencing data from antemortem and postmortem blood samples obtained through the GTEx project and identified extensive time-dependent transcriptomic changes after death [7]. Zhu et al. further demonstrated that PMI-associated expression changes in whole-blood samples may be influenced not only by tissue- and transcript-specific characteristics but also, for certain genes, by genetic variation [9]. Taken together, these studies indicate that the blood transcriptome contains information associated with PMI, although interindividual and biological variability may complicate the interpretation of this relationship [7,9].

The longitudinal study by Antiga et al., which analyzed serial postmortem blood samples from individuals with known times of death, provided evidence for the potential utility of the blood thanatotranscriptome in PMI estimation [11]. The study identified groups of transcripts exhibiting increasing or decreasing abundance over time and reported that pathways associated with cell survival and DNA damage response and repair were prominent during the early postmortem period [11]. The authors interpreted these changes as consistent with the persistence of regulated postmortem processes [11].

In the same study, elastic net-based multi-transcript models were developed, and a root mean square error (RMSE) of 4.75 h was reported for a 30-transcript model using 10-repeat five-fold cross-validation [11]. However, the model tended to overestimate lower PMI values and underestimate values above 20 h. Furthermore, cross-validation does not substitute for independent external validation [11].

Taken together, the available human blood studies support the potential of postmortem transcript profiles to provide quantitative information relevant to PMI estimation. However, given the variability in RNA integrity, transcript-specific kinetics, interindividual and genetic differences, and environmental factors, a universally applicable blood-based transcriptomic model cannot yet be established [7,9,11,20,21]. Before these approaches can be translated into routine forensic practice, these models require external validation in large, independent human cohorts encompassing diverse causes of death, PMI ranges, and environmental conditions.

### 5.2. Liver

The liver is among the tissues investigated in thanatotranscriptomic research because of its tissue-specific transcriptomic characteristics. Studies of human cadaveric liver tissue suggest that the postmortem transcriptome may have potential both for characterizing molecular changes after death and for determining tissue origin [6,22].

In a study by Javan et al. examining human cadaveric liver tissue over a PMI range of 6–48 h, postmortem expression profiles of apoptosis-related genes were evaluated, and mRNA was reported to remain detectable for up to 48 h [6]. Decreased expression of *BCL2* and *BAG3* and increased expression of *CASP3*, *CASP4*, and *CASP9* were observed, with a marked increase in *XIAP* expression [6]. These findings support the view that postmortem RNA changes in the liver do not follow a unidirectional and uniform degradation pattern across all transcripts.

A distinct forensic application of the liver thanatotranscriptome was investigated in a targeted RNA-sequencing study by Javan et al. [22]. The postmortem detectability of eight liver-specific RNA biomarkers, including *AMBP* and *AHSG*, was evaluated in 27 human cadaveric liver samples with PMIs ranging from approximately 3.5 h to 37 days. The findings demonstrated that tissue-specific transcriptomic signals may persist across a broad postmortem interval [22]. The study did not develop a direct model for PMI estimation; rather, it primarily demonstrated the potential utility of RNA biomarkers for postmortem tissue identification.

Taken together, these two studies indicate that the liver thanatotranscriptome may provide information on both time-dependent changes in transcript abundance and tissue-specific expression patterns that can assist in postmortem tissue identification [6,22]. This distinction is important for forensic applications: stable transcripts useful for tissue identification do not necessarily share the same analytical characteristics as time-sensitive biomarkers required for PMI estimation. Therefore, the selection of biomarkers for forensic applications involving the liver transcriptome should be guided by the intended purpose, and their performance should be validated in independent human cohorts.

### 5.3. Prostate

The prostate is of particular interest in studies of postmortem RNA stability and the thanatotranscriptome because it is among the internal organs that undergo relatively delayed decomposition in human remains [10,23]. Tolbert et al. examined the expression of 84 apoptosis-related genes in prostate tissue obtained from five human cadavers using a PCR array and demonstrated that mRNA transcripts remained detectable for up to 5 days (120 h) after death [23].

The study reported significant time-dependent increases in transcripts of the anti-apoptotic genes *BAG1* and *BCL2* and the apoptosis inhibitor *XIAP*, whereas no significant increase was observed for the pro-apoptotic genes *TP53* and *TNFSF10* [23]. In addition, several genes associated with the induction and positive regulation of apoptosis, particularly caspases, exhibited higher transcript levels at 38 h than at 5 days. The authors interpreted these findings as suggesting that active gene expression may persist after death [23].

In a more recent study, Javan et al. used total RNA sequencing to investigate PMI-associated mRNA and long non-coding RNA (lncRNA) expression, as well as alternative splicing events, in human cadaveric prostate tissue [10]. Differential expression patterns and alternative splicing events were identified between high- and low-PMI samples. In the high-PMI group, changes were reported in several biological pathways, including Forkhead box O (FoxO) signaling, ferroptosis, the proteasome, and the citric acid cycle [10]. The authors interpreted these findings as suggesting that transcriptional mechanisms may remain active for up to 5 days after death [10].

Taken together, these studies indicate that prostate tissue may retain measurable RNA profiles at relatively advanced postmortem intervals and may therefore have potential for investigating PMI-associated transcriptomic changes [10,23]. However, the fact that both studies were based on only five cadavers represents an important limitation. The available evidence does not yet support a validated prostate-based model for PMI estimation; therefore, the identified transcriptomic patterns require validation in larger, independent human cohorts encompassing diverse causes of death, environmental conditions, and PMI ranges.

### 5.4. Brain

Brain tissue is of particular interest in postmortem transcriptomic research because of its anatomical and cellular heterogeneity. Given that different brain regions vary in cellular composition, metabolic characteristics, and baseline gene expression profiles, the central nervous system should not be regarded as a homogeneous tissue when evaluating postmortem RNA changes.

The analysis of GTEx data by Zhu et al. demonstrated that PMI-associated gene expression changes are tissue- and gene-specific and that postmortem expression patterns may vary across different brain regions [9]. These findings suggest that the anatomical site of sampling is an important variable when evaluating RNA-based PMI biomarkers in brain tissue.

Time-dependent transcriptomic changes in human brain tissue were investigated in greater detail by Dachet et al. using RNA sequencing (RNA-seq) [24]. In a simulated PMI model in which surgically resected human neocortical tissue was maintained under controlled conditions for 0–24 h, transcripts associated with neuronal activity showed a rapid and selective decline, whereas genes associated with astrocytes and microglia exhibited relative increases in expression over time [24]. In contrast, several commonly used reference genes remained relatively stable. These findings support the view that changes in the postmortem brain transcriptome do not simply reflect uniform RNA degradation but instead exhibit cell type- and transcript-specific kinetics. However, because the study was based on an ex vivo model using surgically resected living human brain tissue, the generalizability of these findings to real-world postmortem forensic cases is limited [24].

The potential of brain tissue for PMI estimation has also been investigated in experimental animal models. Peng et al. evaluated the hypoxia- and apoptosis-related mRNA transcripts *HIF2α*, *HAF*, *AIF*, and *FIH* in mouse brain and heart tissues over a postmortem interval of 0–48 h using quantitative polymerase chain reaction (qPCR) [12]. In brain tissue, *HAF* mRNA abundance was reported to increase after death, and its association with PMI varied depending on the normalization approach employed [12]. Time-dependent changes were also identified in other target transcripts, supporting further investigation of multi-transcript approaches rather than relying on a single RNA marker.

Taken together, these studies indicate that postmortem transcriptomic changes in brain tissue depend on the anatomical region, cell type, target transcript, and normalization method employed [9,12,24]. However, because part of the available evidence is derived from ex vivo human tissue or experimental animal models, the direct forensic applicability of these findings remains limited. To establish the performance of brain-derived RNA biomarkers for PMI estimation, validation is required in large, independent human postmortem cohorts using anatomically standardized sampling protocols and accounting for cause of death, agonal processes, temperature, and storage conditions.

The sample characteristics, tissues examined, postmortem intervals, molecular methods, and principal findings of selected primary studies in the field of thanatotranscriptomics are summarized in Table 1.

**Table 1:** Sample characteristics, tissues examined, postmortem intervals, molecular methods, key findings, potential forensic applications, and major limitations of selected primary studies in the field of thanatotranscriptomics.

Study	Model/Sample	Tissue	PMI/Experimental Duration	Molecular Method	Key Findings	Potential Forensic Application	Major Limitation
Javan et al., 2015 [6]	Human; liver samples from 4 cadavers.	Liver	6–48 h	Apoptosis-specific PCR array	PCR-array analysis of 84 apoptosis-related genes revealed distinct postmortem expression patterns. Seventeen genes were down-regulated, including *BAG3* and *BCL2*, whereas several apoptosis-related genes, including *CASP3, CASP4, CASP7*, and *CASP9*, showed increased expression.	Characterization of postmortem biological processes.	Limited sample size; no directly validated PMI estimation model.
Pozhitkov et al., 2017 [8]	Zebrafish and mouse	Multiple tissues	Species-specific postmortem time series	Transcriptomic analysis	Significantly increased postmortem transcript abundance was identified in 548 zebrafish and 515 mouse transcript profiles relative to live controls. The affected transcripts included genes involved in stress, immunity, inflammation, apoptosis, and developmental processes.	Investigation of the biological basis of postmortem transcriptomic dynamics.	Animal models; direct generalizability to human postmortem tissues is limited.
Zhu et al., 2017 [9]	Human; 316 donors, 2016 samples	15 tissue types/subtypes	1–27 h	Systematic analysis of *GTEx* transcriptomic data	At an FDR of 5%, 7546 distinct PMI-associated genes were identified in at least one of 15 tissues, with the number of PMI-associated genes ranging from 0 to 2763 across tissues. PMI-associated expression changes were tissue-, gene-, and genotype-dependent.	Identification of tissue-specific gene expression patterns associated with PMI.	Retrospective data; substantial tissue-specific and interindividual heterogeneity.
Ferreira et al., 2018 [7]	Human; *GTEx*, 540 donors, 7105 samples	36 tissues	17–1739 min	RNA-seq; machine learning	PMI-associated gene expression changes were strongly tissue-specific, ranging from no significantly changing genes in some tissues to >600 genes in muscle and transverse colon. After adjustment for covariates, an average of 54 genes per tissue (0.2%) showed a significant correlation between expression and PMI. Tissue-specific machine-learning models were developed to predict PMI.	Tissue-specific PMI modeling.	Retrospective GTEx data; dependence on tissue type and preanalytical conditions.
Tolbert et al., 2018 [23]	Human; 5 cadavers	Prostate	Up to 5 days	Apoptosis-related PCR array	Postmortem prostate mRNA remained detectable for up to 5 days. *BAG1, BCL2*, and *XIAP* showed significantly increased expression in a time-dependent manner, whereas *TP53* and *TNFSF10* were not significantly up-regulated.	Assessment of the transcriptome at extended postmortem intervals.	Very small sample size; no independent validation.
Peng et al., 2020 [12]	Mouse model; 29 postmortem time points.	Brain and heart	0–48 h	qPCR; Caspase-3 DNA and 18S normalization.	*HAF* mRNA quantity increased after death in brain tissue with both 18S and Caspase-3 DNA normalization and was significantly correlated with PMI over 0–48 h. PMI-associated degradation patterns of *HIF2α, AIF*, and *FIH* varied according to tissue and normalization method.	Candidate mRNA panels and PMI modeling.	Animal model; findings have not been validated in human samples.
Na, 2020 [25]	Human; 71 autopsy cases	Patella (bone)	1 day–2 years	RT-qPCR; Ce_miR-39_1 spike-in normalization	Expression levels of let-7e and miR-16 decreased with increasing PMI. Both miRNAs showed significant differences between the <1-month PMI group and the longer-PMI groups.	miRNA-based assessment, particularly over extended PMI ranges.	Limited number of target miRNAs; broader independent validation is required.
Antiga et al., 2021 [11]	Human; 54 longitudinal postmortem blood samples from 7 individuals.	Blood	2 h 21 min–37 h 50 min.	Transcriptomic analysis; multivariable modeling	A total of 99 up-regulated and 89 down-regulated transcripts were identified by both ANOVA-Dunnett and linear regression analyses. An elastic net model based on 30 transcripts achieved a cross-validated RMSE of 4.75 h.	PMI estimation.	No independent external validation; the model tended to overestimate lower PMI values and underestimate PMI values above 20 h.
Dachet et al., 2021 [24]	Human; ex vivo PMI simulation using surgically resected neocortical tissue	Brain/neocortex	0–24 h at 24 °C	RNA-seq and histological analysis	Neuronal activity-associated transcripts progressively decreased, whereas astroglial- and microglial-associated gene expression increased over the simulated postmortem interval, with increases continuing up to 24 h.	Characterization of cell type- and transcript-specific postmortem changes in the brain.	Not a real-world serial cadaveric PMI cohort; ex vivo model.
Gupta et al., 2026 [19]	Human; 600 tissue samples yielding 14,400 Ct values.	Heart, liver, lung	Study-specific postmortem time series	RT-qPCR; mRNA, rRNA, and miRNA	Postmortem RNA degradation patterns varied across RNA species and tissues. Multivariable PMI models showed R^2^ values of 0.75 for heart, 0.739 for liver, and 0.57 for lung.	Tissue-specific PMI estimation using multiple RNA markers.	Controlled environmental conditions; validation in independent real-world forensic cohorts is required.
Cianci et al., 2026 [26]	Porcine; 9 postmortem hearts	Heart	0–120 h at 4 °C	RT-qPCR; raw Ct, ΔCt, and ΔΔCt analyses	*HPRT1* and *HMOX1* showed the strongest temporal associations with PMI. Raw Ct-based models outperformed ΔCt-based models. A two-gene *HMOX1/HPRT1* model achieved MAE ≈ 16.4 h and R^2^ ≈ 0.77 in leave-one-heart-out validation.	RT-qPCR-based PMI estimation using cardiac gene-expression models.	Small sample size; controlled refrigerated porcine model; no external validation in independent human forensic cohorts.

*Note:* The table focuses on primary studies; systematic reviews were not included.

## 6. Thanatotranscriptomics and Postmortem Interval Estimation

One of the principal potential applications of thanatotranscriptomics in forensic medicine is postmortem interval (PMI) estimation. RNA-based approaches to PMI estimation generally follow two main strategies. The first examines the postmortem stability and degradation kinetics of specific transcripts. The second evaluates multiple transcripts simultaneously to develop multivariable models for PMI estimation [4,5,7,9,11,12]. Whereas the first approach focuses on the time-dependent behavior of selected RNA markers, the second aims to leverage the multidimensional nature of the postmortem transcriptome.

Multi-transcript models have the potential to reduce some of the limitations associated with the susceptibility of individual transcripts to biological and environmental variability. However, simply increasing the number of transcripts included in a model does not necessarily improve predictive performance. Particularly in high-dimensional transcriptomic datasets with limited sample sizes, variable selection, overfitting, model calibration, and independent external validation are critical methodological considerations. Model performance should therefore be evaluated not solely on the basis of goodness-of-fit measures, but also by considering prediction error and results obtained in independent datasets.

Ferreira et al. analyzed 7105 samples from 540 donors across 36 human tissues within the GTEx project and demonstrated that postmortem gene expression changes are markedly tissue-specific [7]. The investigators also developed machine-learning models for PMI estimation using transcriptomic profiles and reported that accessible tissues, such as skin and subcutaneous adipose tissue, may provide informative signals for estimating time since death [7]. Similarly, transcript-based and multi-RNA-marker models developed in other human studies and experimental models support the potential of RNA profiles to provide quantitative information associated with PMI [11,12,17,18,19].

Nevertheless, the generalizability of current models remains a major concern. In addition to tissue type and anatomical sampling site, temperature, cause of death and agonal processes, PMI range, interindividual biological variability, sampling and storage conditions, and RNA isolation and normalization methods may influence model performance [4,5,7,9,16]. Therefore, a model demonstrating high performance in a particular dataset cannot be assumed to achieve comparable predictive accuracy when applied to independent forensic cases.

Thanatotranscriptome-based PMI estimation should therefore be conceptualized not as a single, universal “molecular clock,” but rather as an approach that integrates tissue- and transcript-specific postmortem kinetics within multivariable models. Future models should define not only which transcripts are associated with PMI, but also the tissues, PMI ranges, and environmental conditions under which these associations remain reliable. Standardized sampling and analytical protocols, explicit reporting of prediction uncertainty, and external validation in large, independent human cohorts are essential prerequisites for translating these models into routine forensic practice.

## 7. Use of mRNA in PMI Estimation

Messenger RNA (mRNA) is among the most extensively investigated RNA species in postmortem molecular research and has been evaluated as a potential biomarker, particularly for the estimation of early postmortem intervals [4]. mRNA-based studies encompass a range of approaches, from targeted measurement of time-dependent changes in specific transcripts to large-scale transcriptomic analyses.

Quantitative reverse transcription polymerase chain reaction (RT-qPCR) is one of the most commonly used methods for mRNA analysis and enables quantitative assessment of selected transcript levels. RNA sequencing (RNA-seq), in contrast, allows the simultaneous analysis of large numbers of transcripts and the characterization of postmortem expression patterns on a broader scale [4,7]. However, because deterioration in RNA integrity can affect quantitative gene expression measurements, postmortem mRNA data should be interpreted in conjunction with RNA quality [16].

According to the systematic review by Cianci et al., *ACTB* and *GAPDH* are among the most frequently investigated mRNA markers in studies of PMI estimation [4]. However, the postmortem behavior of these transcripts may vary depending on tissue type, temperature, and experimental conditions. Therefore, an association between a particular mRNA transcript and PMI observed in one tissue or under specific experimental conditions does not guarantee that the same relationship will be maintained under different conditions [4].

Normalization represents one of the major methodological challenges in mRNA-based PMI studies. In a study comparing potential endogenous reference genes in human postmortem tissues, Koppelkamm et al. demonstrated that commonly used reference genes require specific validation under postmortem conditions [27]. Similarly, Zhang et al., who evaluated heart, brain, kidney, and skin tissues from 109 human autopsy cases, demonstrated that reference gene stability is tissue-dependent [28]. *GAPDH*, U6, and 18S rRNA showed broader utility under the study conditions, whereas the reliability of *ACTB* and *B2M* was limited to cardiac tissue [28].

These findings indicate that commonly used genes such as *ACTB* and *GAPDH* should not automatically be assumed to represent stable reference genes in postmortem studies [4,27,28]. Reference genes used for normalization should be validated for the specific tissue, PMI range, and experimental conditions under investigation, and, whenever possible, multiple candidate reference genes should be systematically compared. Inappropriate normalization strategies may lead to misinterpretation of genuine postmortem changes in target transcripts and reduce the reliability of developed PMI models.

## 8. Use of miRNA in PMI Estimation

Thanatotranscriptomic research is not limited to mRNA. In recent years, microRNAs (miRNAs) have also been investigated as potential biomarkers for PMI estimation [29,30]. miRNAs are small non-coding RNA molecules, approximately 18–24 nucleotides in length, that play a role in the post-transcriptional regulation of gene expression. The relatively high stability of certain miRNAs under postmortem conditions has made these molecules of particular interest for forensic research [29,30]. Compared with mRNA, miRNAs may offer greater postmortem stability because of their small size and resistance to degradation; however, this relative advantage is not uniform across all miRNAs or tissues [29,30].

In a systematic review encompassing 18 studies, Pasaribu et al. reported that certain miRNAs remain measurable during the postmortem period and may exhibit PMI-associated changes [29]. Similarly, a more recent systematic review by Cianci et al. indicated that miRNAs may have potential for both PMI estimation and normalization in postmortem RNA analyses, although their stability may vary according to the specific miRNA and tissue examined [30].

Primary human data also support this potential. Na investigated let-7e and miR-16 levels in 71 patellar samples obtained from autopsy cases with PMIs ranging from 1 day to 2 years and reported that the expression levels of both miRNAs were negatively correlated with increasing PMI [25]. The identification of significant differences, particularly between cases with PMIs of less than 1 month and those with longer PMIs, suggests that miRNAs in bone tissue may also serve as potential biomarkers over extended postmortem intervals [25].

More recently, Gupta et al. jointly evaluated the degradation of mRNA, rRNA, and miRNA in human heart, liver, and lung tissues and reported that miR-122 and miR-1 may have potential for PMI estimation because of their relatively stable postmortem behavior [19]. These findings further support the tissue-specific nature of miRNA behavior and suggest that integrating different RNA markers, rather than relying on a single RNA species, may improve approaches to PMI estimation [19].

Large-scale miRNA profiling approaches have also contributed to the identification of candidate biomarkers. Lee et al. identified 154 differentially expressed miRNAs in mouse myocardial tissue using high-throughput sequencing and subsequently validated nine apoptosis-related miRNAs by qRT-PCR [31]. Although significant linear relationships were identified between the levels of several miRNAs and PMI, the use of an experimental animal model limits the applicability of these findings to human forensic cases [31].

**Figure 1:**
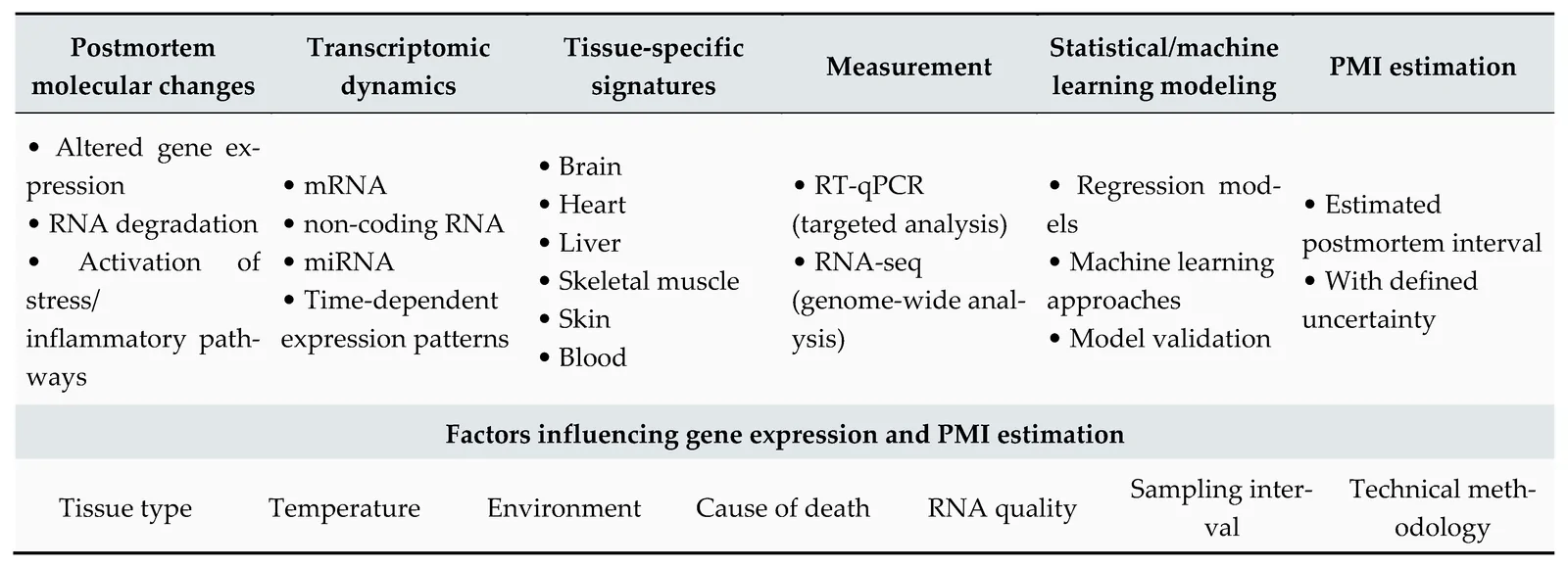
Schematic overview of thanatotranscriptomic approaches for postmortem interval estimation.

Nevertheless, postmortem miRNA stability cannot be generalized across all miRNAs and tissues. In addition to tissue type, cause of death, environmental conditions, and other biological variables, differences in sampling, storage, normalization, and analytical methods may influence the observed results [29,30]. Analytically, miRNA assessment also requires methodological approaches appropriate for short RNA molecules, including suitable RNA isolation, assay selection, and normalization strategies, whereas mRNA-based analyses involve distinct transcript-specific considerations; these methodological differences should be considered when comparing their potential for PMI estimation [29,30]. Therefore, miRNAs cannot currently be regarded as independently validated biomarkers for PMI estimation. Despite differences in their molecular stability and analytical characteristics, both mRNA- and miRNA-based approaches still require further standardization and independent validation before their routine application to forensic PMI estimation. The development of tissue-specific miRNA panels and their integration with other transcriptomic markers, such as mRNA, in multivariable models represents a promising direction for future research. Translation of such models into routine forensic practice will require standardized protocols and external validation in independent human cohorts. The overall conceptual framework of thanatotranscriptomic approaches to PMI estimation, from postmortem molecular changes to transcriptomic measurement and statistical modeling, is summarized in Figure 1.

## 9. Molecular Methods Used in Thanatotranscriptomic Studies

The selection of molecular methods in thanatotranscriptomic studies depends on several factors, including the study objective, the RNA species of interest, sample characteristics, and RNA quality. Among the available approaches, RT-qPCR and RNA sequencing (RNA-seq) are the principal molecular methods used to investigate postmortem transcriptomic changes [4,7,32].

### 9.1. RT-qPCR

Reverse transcription quantitative polymerase chain reaction (RT-qPCR) is a highly sensitive method that enables the targeted and quantitative assessment of predefined RNA transcripts. Accordingly, it is widely used to evaluate and validate candidate transcripts associated with postmortem interval (PMI) [4,25]. However, an important limitation of the method is that it is restricted to preselected target transcripts. In addition, the reliability of the resulting measurements may be influenced by preanalytical and analytical factors, including RNA integrity, primer design, reference gene selection, and normalization strategy [4,16].

Peng et al. [12] demonstrated in an experimental study that the relationship between target transcripts and PMI may vary depending on the normalization approach employed. This finding indicates that the analytical reliability of RT-qPCR-based PMI studies depends not only on the selection of appropriate target transcripts but also on the selection of reference genes and the standardization of normalization strategies.

### 9.2. RNA Sequencing (RNA-Seq)

RNA sequencing (RNA-seq) enables the simultaneous, large-scale analysis of numerous transcripts and therefore represents an important tool for characterizing the postmortem transcriptome and identifying novel candidate biomarkers. Large-scale studies using data from the Genotype-Tissue Expression (GTEx) project have demonstrated the value of RNA-seq for identifying the tissue- and gene-specific characteristics of postmortem gene expression changes [7,9].

The transcriptome-wide and hypothesis-independent analytical capacity of RNA-seq enables comprehensive molecular profiling without requiring the prior selection of candidate transcripts. However, differences in sample and library preparation, sequencing parameters, bioinformatic data-processing pipelines, and normalization approaches may affect comparability across studies. Therefore, although RNA-seq represents a powerful approach, particularly for the discovery of novel candidate biomarkers, biomarkers identified through transcriptome-wide analyses should be validated in independent sample sets, preferably using targeted molecular methods.

### 9.3. Normalization and Assessment of RNA Quality

The use of appropriate normalization strategies and the assessment of RNA quality are critical for obtaining reliable and reproducible quantitative results in postmortem RNA analyses. Because the stability of transcripts used as references may vary according to tissue type and postmortem conditions, commonly used reference genes should not be assumed to be inherently stable in postmortem samples [4,27,28]. Reference gene stability should therefore be validated under the specific tissue and experimental conditions under investigation.

RNA integrity is another important factor that may influence measured gene expression levels and their interpretation. Studies of human postmortem tissues have demonstrated that RNA integrity may vary across tissues and that RNA degradation can affect the interpretation of results obtained by RT-qPCR [16]. However, overall RNA integrity and the postmortem stability of a specific transcript represent distinct concepts. RNA quality metrics should therefore be interpreted in conjunction with the time-dependent behavior of target transcripts and the characteristics of the analytical method employed [5,16].

In summary, RNA-seq and RT-qPCR play complementary roles in thanatotranscriptomic research: RNA-seq facilitates large-scale biomarker discovery and characterization of the postmortem transcriptome, whereas RT-qPCR enables targeted quantitative assessment and validation of selected candidate transcripts. For both approaches, standardized sampling, appropriate assessment of RNA quality, reliable normalization strategies, and independent validation are essential for ensuring the reproducibility of findings and their translation into forensic practice.

## 10. Potential Forensic Applications of the Thanatotranscriptome

In addition to postmortem interval (PMI) estimation, thanatotranscriptomic analyses have potential applications in addressing other forensic questions, including the determination of tissue origin and the molecular characterization of biological processes associated with death. However, most of these applications remain at the research stage and require methodological standardization and independent validation before they can be translated into routine forensic practice.

### 10.1. Postmortem Interval (PMI) Estimation

PMI estimation is one of the most extensively investigated forensic applications of the thanatotranscriptome. Statistical and machine-learning models incorporating time-dependent changes in postmortem transcript abundance and RNA degradation may enable quantitative estimation of PMI [4,7,11]. Although studies of human blood and various human tissues support the feasibility of this approach, the dependence of current models on tissue type, the PMI range examined, sample characteristics, and study conditions limits their generalizability [7,9,11]. Therefore, before these models can be implemented in routine forensic practice, they should be externally validated in independent cohorts using standardized protocols, with performance reported across predefined PMI ranges and accompanied by appropriate measures of predictive uncertainty, including confidence or prediction intervals, as appropriate.

### 10.2. Tissue and Organ Identification

Tissue-specific postmortem transcript profiles may potentially be used for the molecular determination of the tissue origin of biological samples. Javan et al. demonstrated that tissue-specific transcriptomic biomarkers could be detected in human cadaveric liver tissue even at extended postmortem intervals [22]. This approach may provide complementary molecular information, particularly in samples in which morphological assessment is complicated by autolysis or advanced postmortem changes. However, before transcriptomic markers for tissue identification can be translated into forensic practice, they require validation in independent sample sets encompassing different tissue types and postmortem conditions.

### 10.3. Molecular Information Related to Cause and Mechanism of Death

The postmortem transcriptome may reflect molecular changes associated with biological responses occurring during the dying process, including hypoxia, ischemia, inflammation, cellular stress, and apoptosis [6,23,32]. Postmortem gene expression profiles may therefore provide complementary molecular information for the evaluation of cause and mechanism of death, particularly in cases in which morphological findings are limited or nonspecific.

Primary human studies support this potential. González-Herrera et al. evaluated the mRNA levels of *TNNI3*, *MYL3*, *TGFB1*, *MMP9*, and *VEGFA*, which are associated with ischemia and myocardial injury, in myocardial tissue, pericardial fluid, and blood samples obtained from 30 cadavers with different causes of death [33]. Differences in the expression of certain transcripts among cause-of-death groups, including sudden cardiac death and mechanical asphyxia, suggest that postmortem mRNA analysis may provide complementary molecular information, particularly for the assessment of early myocardial ischemia [33].

Similarly, Schober et al. compared mRNA and miRNA expression profiles in the cerebellum between cases of death following severe frontal cortex injury and cases of natural death and identified differential expression patterns associated with hypoxia [34]. Increased expression of *FOSB* and *IL6* and decreased expression of *HSD11B1* and *HSPA12B* were observed, and certain combinations of mRNA and miRNA markers were reported to discriminate between the study groups [34]. These findings support the possibility that hypoxic and cellular stress responses occurring during the dying process may be reflected in postmortem transcript profiles.

However, these molecular changes should not be assumed to be specific to a particular cause of death. Hypoxia, ischemia, inflammation, cellular stress, and apoptosis are biological responses that may occur across different mechanisms and causes of death. In addition, antemortem diseases, agonal processes, therapeutic interventions, and postmortem changes may influence measured transcript levels. Therefore, neither a single transcript nor a transcriptomic profile should be interpreted as independently establishing a specific cause of death. Importantly, statistically significant differences in transcript expression between cause-of-death groups should be distinguished from biomarkers with demonstrated diagnostic performance; establishing such performance requires appropriate measures of discrimination and validation in independent cohorts.

A more reliable approach is to interpret thanatotranscriptomic findings in conjunction with autopsy findings, histopathology, toxicology, and other case-specific forensic evidence. In this context, the thanatotranscriptome should be regarded not as an independent diagnostic tool replacing conventional forensic pathology methods, but as a source of complementary molecular information that may enhance their interpretation.

## 11. Limitations of the Thanatotranscriptome

Although the thanatotranscriptome has considerable potential, particularly for postmortem interval (PMI) estimation, current approaches have not yet been sufficiently standardized and validated for independent use in routine forensic practice [4,5]. Environmental, biological, preanalytical, and analytical variables affecting postmortem RNA profiles limit the accuracy, reproducibility, and generalizability of current models.

### 11.1. Temperature, Sampling, and Storage Conditions

Temperature is one of the principal environmental factors affecting postmortem RNA stability and degradation kinetics [4,5,15]. Consequently, the direct application of PMI models developed under controlled experimental conditions to cases involving different environmental conditions may lead to inaccurate or misleading estimates [4,5].

The PMI should also be distinguished from the preanalytical interval between sample collection and laboratory analysis. In addition to the interval from death to sampling, post-collection transport and storage conditions, as well as the time elapsed before RNA isolation, may influence the measured RNA profile [4,5,7]. Standardization and detailed reporting of these variables are important for ensuring comparability across studies and the reproducibility of developed models.

### 11.2. Tissue and Transcript Heterogeneity

Postmortem RNA behavior varies across both tissues and transcripts [4,5,9]. The same transcript may exhibit different postmortem kinetics across different tissues, while the stability and degradation rates of individual transcripts within the same tissue may also vary [9].

This heterogeneity complicates the development of a single universal transcriptomic model for PMI estimation that remains applicable across different tissue types and broad PMI ranges. Therefore, the development of tissue-specific transcript panels and models validated for defined PMI ranges appears to be a more feasible approach.

### 11.3. Antemortem and Interindividual Biological Variables

Cause of death and the agonal process may influence the baseline transcriptomic profile at the time of death. The molecular effects of antemortem and perimortem processes, including hypoxia, ischemia, inflammation, and cellular stress, may be reflected in transcript levels measured in postmortem samples [32,33,34]. Therefore, the postmortem RNA profile should not be regarded solely as a function of PMI.

Interindividual biological variability represents another factor that may influence model performance. Zhu et al. demonstrated genotype-by-PMI interactions for certain PMI-associated gene expression changes [9]. This finding supports the need to consider individual biological characteristics as potential confounding variables and to validate these models in independent and heterogeneous human cohorts.

### 11.4. Advanced Postmortem Changes

As PMI increases, progressive autolysis and RNA degradation may complicate molecular analyses; however, these processes do not occur at the same rate across all tissues and transcripts [4,5]. The persistence of certain transcripts even at extended postmortem intervals indicates that the mere presence or absence of a transcript cannot be interpreted as a reliable indicator of PMI [22].

Accordingly, models developed for the early postmortem period should not be directly extrapolated to extended postmortem intervals or stages of decomposition. For each model, the applicable tissue type, environmental conditions, and particularly the validated PMI range should be clearly defined.

### 11.5. Lack of Standardization and External Validation

One of the major barriers to translating thanatotranscriptomic research into routine forensic practice is methodological heterogeneity. Differences in sampling procedures, tissue selection, RNA isolation, RNA quality assessment, normalization strategies, and analytical platforms complicate direct comparisons across studies and limit the reproducibility of findings [4,5,16,27,28].

Another important limitation is the lack of independent external validation. High performance achieved by a model developed and cross-validated within the same dataset does not guarantee comparable predictive accuracy in independent forensic cases. Antiga et al. reported a root mean square error (RMSE) of 4.75 h for a model based on 30 RNA transcripts for postmortem intervals of up to 38 h in human postmortem blood [11]. However, the authors specifically emphasized the need for further validation in independent sample sets before the model could be applied to forensic cases [11].

Model performance should be evaluated not only by using correlation or goodness-of-fit measures but also by reporting prediction errors and metrics that quantify predictive uncertainty, thereby allowing a more robust assessment of performance under real-world forensic conditions. Furthermore, a substantial proportion of the current literature is based on animal models, whereas studies involving human postmortem samples remain relatively limited, restricting the direct generalizability of experimental findings to forensic cases [5].

## 12. From Single Biomarkers to Multi-Transcript Models

The variability of postmortem RNA behavior across tissues, transcripts, and environmental conditions complicates the use of a single biomarker for PMI estimation. Therefore, the combined evaluation of multiple transcripts may enable the development of more comprehensive transcriptomic signatures that capture different molecular features of the postmortem process [7,9,11].

Multi-transcript models may integrate different mRNA and miRNA markers with measures of transcript stability or degradation; in appropriate datasets, PMI-related environmental variables such as temperature may also be incorporated into the model. PMI estimation can thus be conceptualized as a multivariable prediction problem integrating multiple molecular and environmental variables, rather than as an approach based solely on a linear relationship between a single transcript and time since death.

Available human studies demonstrate the feasibility of this approach. Antiga et al. developed a PMI estimation model using elastic net regression to integrate multi-transcript data from human postmortem blood [11]. Ferreira et al. used gradient boosting-based models to estimate time since death from large-scale human transcriptomic data [7]. These studies demonstrate that postmortem transcriptomic data can be integrated using multivariable computational approaches, thereby extending PMI estimation beyond single-biomarker strategies.

However, increasing the number of variables included in a model does not necessarily improve predictive performance. The risk of overfitting is particularly important in transcriptomic datasets characterized by limited sample sizes and large numbers of variables. Therefore, biologically and statistically informed variable selection and appropriate model validation strategies are essential for reducing overfitting and assessing the reliability of multi-transcript models.

From a forensic perspective, the primary objective should not merely be to develop models that perform well in the training dataset, but to establish models with demonstrated generalizability across diverse individual and environmental conditions and with quantitatively characterized predictive uncertainty.

## 13. Future Directions in Thanatotranscriptomics

A major future objective of thanatotranscriptomic research should be not merely to identify transcripts that change after death, but to translate these biological signals into reliable, reproducible, and generalizable forensic models. Achieving this objective will require a systematic research framework encompassing biomarker discovery, analytical validation, model development, and independent external validation.

One of the principal priorities is the establishment of large, independent human cohorts. Future studies should adequately represent different PMI ranges, causes of death, environmental conditions, and interindividual biological variability. Study designs comparing multiple tissues obtained from the same individual may also help account for interindividual variability and enable a more detailed characterization of tissue-specific postmortem transcriptomic changes.

Standardization is a fundamental prerequisite for translating research findings into forensic practice. Harmonization of sampling, transport, storage, RNA isolation, RNA quality assessment, and normalization procedures is necessary to improve comparability and reproducibility across studies [4,5]. Within this framework, RNA-seq may be used for large-scale biomarker discovery, whereas validation of identified candidates in independent sample sets using targeted methods such as RT-qPCR may provide a complementary pathway toward analytical validation.

Another important direction for future research is the transition from single-transcript approaches to multi-transcript panels. Integrating complementary molecular information derived from different RNA species, such as mRNA and miRNA, with tissue type, temperature, and other relevant variables may better capture the biological heterogeneity of the postmortem process [4,11,29,30]. Although machine learning represents a powerful tool for analyzing high-dimensional transcriptomic data, high model performance alone does not establish forensic validity. Future models should therefore undergo external validation in independent datasets and real-world forensic case series, with their performance evaluated using prediction errors and measures of predictive uncertainty. To support translation into forensic practice, future studies should adopt multicenter designs with standardized preanalytical procedures and predefined development and validation cohorts. In addition, their incremental value should be evaluated by determining whether transcriptome-based models improve PMI estimation when used alongside established forensic methods, rather than assessing their performance only as stand-alone approaches.

From a broader perspective, the integration of transcriptomic data with other molecular data layers, such as proteomics and metabolomics, may enable more comprehensive modeling of postmortem biological changes [35,36]. Although multi-omics approaches may theoretically provide complementary information across different molecular levels, whether they offer incremental predictive value over models based solely on transcriptomic data remains to be demonstrated in independent human studies.

Ultimately, the future of thanatotranscriptomics depends not on the pursuit of a single universal “molecular clock,” but on the development of robust forensic models that account for postmortem biological heterogeneity and demonstrate practical value across real-world forensic settings [4,5].

## 14. Discussion

Current evidence indicates that the postmortem transcriptome cannot be regarded solely as the product of passive and uniform RNA degradation. Although integrated physiological functions cease with organismal death, cellular and molecular processes in different tissues do not terminate simultaneously [3,6,7,8]. The measured postmortem RNA profile may reflect the combined effects of the stability of transcripts synthesized antemortem, selective RNA degradation, and potentially residual transcriptional processes [7,8]. Therefore, an increase in the relative abundance of a transcript during the postmortem period should not, by itself, be interpreted as direct evidence of de novo RNA synthesis.

The study conducted by Pozhitkov et al. in animal models demonstrated that the relative abundance of certain transcripts may increase after death, supporting the view that the postmortem transcriptome cannot be explained solely by uniform RNA degradation [8]. However, because these findings were derived from mouse and zebrafish models, their generalizability to human forensic cases is limited. Human studies, in contrast, have demonstrated marked tissue- and transcript-specificity. Large-scale studies by Ferreira et al. and Zhu et al. showed that postmortem gene expression changes depend on tissue type, gene, and, in some instances, interindividual biological characteristics [7,9]. This heterogeneity complicates the development of a single universal transcriptomic model for PMI estimation that remains applicable across different tissues and postmortem conditions.

Nevertheless, substantial evidence indicates that transcriptomic data may provide quantitative information relevant to PMI estimation. In the longitudinal human blood study by Antiga et al., both increasing and decreasing postmortem transcripts were identified, and an elastic net regression model based on multiple transcripts achieved a root mean square error (RMSE) of 4.75 h using 10-repeat five-fold cross-validation [11]. However, the model tended to overestimate lower PMI values and underestimate values above 20 h, and it was not externally validated in an independent sample set. These limitations indicate that a single overall performance metric is insufficient to establish forensic applicability. Accordingly, model evaluation should consider not only overall prediction error but also error distributions across the PMI range and uncertainty associated with individual predictions.

Similarly, although miRNAs have been investigated as potential PMI biomarkers because of their relatively high stability, their postmortem behavior may vary according to the specific miRNA, tissue type, and study conditions [29,30]. Collectively, these findings support further investigation of tissue-specific panels integrating mRNA and miRNA markers that represent distinct postmortem kinetics, rather than relying on a single transcript.

Biological and environmental variables other than PMI should also be considered when interpreting postmortem RNA profiles. Hypoxia, ischemia, inflammation, and cellular stress responses associated with the cause of death and agonal process may influence the baseline transcriptomic profile at the time of death [32,33,34]. Among environmental factors, temperature warrants explicit consideration in predictive modeling, either as a model variable or through the development and validation of temperature-specific models across different thermal conditions [37]. Disentangling the effects of these factors from PMI-associated molecular signals is substantially more complex in real-world forensic cases than under controlled experimental conditions.

Analytical variability represents another important limitation. RNA integrity may differ across postmortem tissues and can affect the interpretation of RT-qPCR results [16]. Furthermore, commonly used reference genes such as *ACTB* and *GAPDH* should not be assumed to be inherently stable under postmortem conditions; reference genes used for normalization should be validated for the specific tissue and experimental conditions under investigation [4,27,28]. Recent experimental evidence from postmortem cardiac tissue indicates that reference-gene stability and the choice of data representation, including raw Ct and ΔCt/ΔΔCt-based approaches, may influence the predictive performance of RT-qPCR-based PMI models [26].

The computational modeling of multi-transcript data represents an important area of development in thanatotranscriptome-based PMI estimation [7,11]. However, in high-dimensional transcriptomic datasets, a large number of variables relative to sample size may increase the risk of overfitting.

The forensic potential of the thanatotranscriptome extends beyond PMI estimation. The detectability of tissue-specific transcripts at extended postmortem intervals provides a molecular approach to determining tissue origin [22], whereas the assessment of hypoxia, ischemia, apoptosis, and other cellular stress responses associated with the dying process may provide complementary molecular information relevant to the cause and mechanism of death [6,23,33,34]. However, many of these biological responses are not specific to a particular cause of death. Transcriptomic findings should therefore not be interpreted as independent diagnostic evidence but rather integrated with autopsy findings, histopathology, toxicology, and other case-specific forensic evidence.

Overall, the available evidence indicates that the thanatotranscriptome should be regarded not as a precise “molecular clock of death,” but as a complementary molecular approach capable of providing probabilistic and quantitative information relevant to PMI estimation. Accordingly, the principal objective of future research should not merely be to develop models that achieve high performance within a particular training dataset, but to establish transcriptome-based tools with demonstrated generalizability and incremental informational value in real-world forensic cases.

## 15. Conclusions

Thanatotranscriptomic research indicates that postmortem RNA profiles do not merely reflect passive and uniform degradation but may exhibit dynamic changes that vary according to tissue, transcript, and time [3,6,7,8]. Current evidence from human studies supports the potential of these molecular changes to provide quantitative information relevant to PMI and the incorporation of multi-transcript data into PMI estimation models [7,11].

Nevertheless, the thanatotranscriptome should not currently be regarded as a precise “molecular clock” or an independent method for determining PMI. Tissue and transcript heterogeneity, environmental and biological variables, preanalytical and analytical variability, lack of methodological standardization, and limited external validation currently restrict the translation of these approaches into routine forensic practice [4,5].

Accordingly, the most realistic current forensic role of the thanatotranscriptome is not to determine the time of death independently, but to provide a complementary and quantitative layer of molecular information for PMI assessment. Further research should determine whether such models yield reproducible and forensically relevant improvements in PMI assessment across real-world forensic settings.

## Data Availability

No new data were created or analyzed in this study. Data sharing is not applicable to this article.

## Abbreviations

FoxO	Forkhead box O
GTEx	Genotype-Tissue Expression
lncRNA	long non-coding RNA
miRNA	microRNA
mRNA	messenger RNA
PMI	postmortem interval
qPCR	quantitative polymerase chain reaction
RIN	RNA integrity number
RMSE	root mean square error
RNA-seq	RNA sequencing
rRNA	ribosomal RNA
RT-qPCR	reverse transcription quantitative polymerase chain reaction
